# Complete Genome Sequences of Mycobacteriophages Dallas and Jonghyun

**DOI:** 10.1128/MRA.00304-21

**Published:** 2021-07-08

**Authors:** Laricca Y. London, Mary A. Ayuk, Diana Effiom, Folasade Fashina, Briana J. Louis, Sara S. Tolsma, Adrian D. Allen, Leon A. Dickson, Somiranjan Ghosh, Ayele Gugssa, Hemayet Ullah, Glory B. Bassey, Lourds M. Fernando, Madison M. Moore, Jerome J. Oliver, Esohe G. Irabor, Swagota D. Roy, Benedict K. Quagraine, Michael Smith, Howard University SEA-PHAGES Students,, Winston A. Anderson, Courtney J. Robinson

**Affiliations:** a Department of Biological and Environmental Science, Alabama A&M University, Normal, Alabama, USA; b Department of Biology, Howard University, Washington, DC, USA; c Department of Biology, Northwestern College, Orange City, Iowa, USA; Queens College CUNY

## Abstract

Two temperate mycobacteriophages, Dallas and Jonghyun, were isolated from soil in Washington, DC, using the bacterial host Mycobacterium smegmatis mc^2^155. Analysis of the genomes revealed that Dallas and Jonghyun belong to clusters J and G, respectively. The structures of the genomes are typical of their respective clusters.

## ANNOUNCEMENT

Mycobacteriophages Dallas and Jonghyun were isolated from two soil samples in Washington, DC (38.9227N, 77.0209W, and 38.9219N, 77.0186W, respectively), by students investigating mycobacteriophage diversity in the Phage Hunters Advancing Genomics and Evolutionary Science program at Howard University (1). Soil was incubated in 7H9 broth at 37°C for 2 h with shaking, followed by centrifugation and filtration (0.22-μm-pore-size filters) of the supernatant. The filtrate was then incubated aerobically with Mycobacterium smegmatis mc^2^155 at 37°C for 48 h. This was followed by filtration of the enrichment and plating on M. smegmatis mc^2^155. Phage populations were subjected to three rounds of plaque purification, followed by DNA isolation using the Wizard DNA Clean-Up System (Promega) (2). Genomic DNA libraries were generated using NEBNext Ultra II FS kit v3 reagents with dual-indexed barcoding (New England BioLabs). Pooled libraries were run on an Illumina MiSeq sequencer to yield 150-bp single-end reads (Dallas, 1,058,535 bp; Jonghyun, 291,316 bp). The reads were trimmed and assembled using Newbler v2.9. Each case yielded a single contig, which was checked for completeness, accuracy, and phage genomic termini using Consed v29 (3–5). Each genome was annotated using the Phage Evidence Collection and Annotation Network (PECAAN; http://pecaan.kbrinsgd.org) and DNA Master v5.23.3 (cobamide2.bio.pitt.edu/computer.htm). Gene presence, start sites, and functions were determined using Glimmer v3.0, GeneMark v2.5, Starterator v1.1 (https://github.com/SEA-PHAGES/starterator), Phamerator Actino_Draft v402 (https://phamerator.org), BLAST v2.11.0+, PhagesDB, HHpred v3.0, and the NCBI Conserved Domain Database v3.19; tRNAs were detected using ARAGORN v1.2.38 and tRNAscan-SE v2.0 (6–13). Phylogeny was determined using the NCBI Sequence Read Archive (SRA) Taxonomy Analysis Tool (14). Default parameters were used for all software unless otherwise specified. Table 1 provides descriptions of each genome.

**TABLE 1:** Genome characterization of two temperate mycobacteriophages

Phage name	GenBank accession no.	Cluster	Sequencing coverage (×)	Genome size (bp)	G+C content (%)	No. of protein-coding genes	No. of tRNAs
Dallas	MN062701	J	1,353	111,843	60.8	241	1
Jonghyun	MN444875	G	984	41,904	66.6	61	0

Both mycobacteriophages were identified as temperate based on plaque turbidity and the presence of genes associated with lysogeny, such as those that encode tyrosine integrase (SEA_DALLAS_80 and SEA_JONGHYUN_32), the excise protein (SEA_DALLAS_79 and SEA_JONGHYUN_34), and an immunity repressor (SEA_DALLAS_99 and SEA_JONGHYUN_33).

The double-stranded DNA genome of Dallas is linear with a 4-base overhang (ATCC). Actinobacteriophage clusters contain genomes with sequence similarity that is maintained for more than one-half of the length of each of the individual genomes and can be subdivided into subclusters (15, 16). Dallas was assigned to cluster J based on nucleotide similarity to other phages in this cluster (17). These phages are members of the *Siphoviridae* family (17). Similar to other cluster J phages, the genome length of Dallas is 111,843 bp, with one tRNA and 242 predicted protein-coding genes (17).

The genome of Jonghyun has a defined 11-base 3′ overhang (CCCCATGGCAT) and 98.1% of reads affiliated with *Siphoviridae*. The phage was assigned to cluster G, subcluster G1, based on nucleotide similarity to other subcluster G1 phages such as Chance64 (GenBank accession no. MF919497) (100.0%). Based on information in PhagesDB, the 41,904-bp genome size is typical of subcluster G1 (12).

### Data availability.

The complete genome sequences of phages Dallas and Jonghyun are available in GenBank (accession no. MN062701 and MN444875, respectively). The raw sequencing reads are available in the NCBI SRA under accession no. SRX10013859 and SRX10013862, respectively.
